# Multiomic analysis of dark tea extract on glycolipid metabolic disorders in db/db mice

**DOI:** 10.3389/fnut.2022.1006517

**Published:** 2022-09-08

**Authors:** Caiqiong Wang, Minghai Hu, Yuhang Yi, Xinnian Wen, Chenghao Lv, Meng Shi, Chaoxi Zeng

**Affiliations:** ^1^Laboratory of Food Function and Nutrigenomics, College of Food Science and Technology, Hunan Agricultural University, Changsha, China; ^2^Department of Neurobiology and Human Anatomy, School of Basic Medical Science, Central South University, Changsha, China; ^3^College of Bioscience and Biotechnology, Hunan Agricultural University, Changsha, Hunan, China

**Keywords:** dark tea, glycolipid metabolism, gene expression, gut microbiota, multiple omics

## Abstract

Glycolipid metabolic disorder is a serious threat to human health. Dark tea is a kind of traditional Chinese tea, which may regulate the glycolipid metabolic disorders. Dark tea extract (DTE) is the water extraction obtained from dark tea. Compared with traditional dark tea, DTE has the benefits of convenient consumption and greater potential for promoting health. However, the regulation of DTE on glycolipid metabolism and its molecular mechanism is rarely investigated. In our study, DTE was used as raw material to study the effect and molecular mechanism of its intervention on the glycolipid metabolic in db/db diabetic mice by using multiomics analysis and modern biological techniques. (1) DTE could significantly reduce fasting glucose in diabetic db/db mice, and the higher dose group has a better effect. Histopathological examination showed that DTE slightly improve the number of islets and decrease the number of islet β cells in the pancreatic tissue in db/db mice. (2) RNA-Seq was used to analyze the gene expression in liver tissue. In terms of biological processes, DTE mainly affected the inflammation and fatty acid metabolism. In terms of cell components, the lipoprotein and respiratory chain are mainly affected. In the aspect of molecular function, DTE mainly affected the redox related enzyme activity, iron ion binding and glutathione transferase. Arachidonic acid metabolism pathway, glutathione metabolism and PPAR signaling pathway were enriched by DTE with the results of KEGG pathway enrichment. In addition, real-time PCR results confirmed that DTE could significantly activate key genes of PPAR signaling pathway like *Fabp1, Cyp4a1, Ehhadh, Cyp4a32, Aqp7* and *Me1*. (3) 16s rDNA showed that DTE could significantly decrease the ratio of *Firmicutes/Bacteroidetes* and the abundance of *Proteobacteria*, and increased the relative abundance of *Verrucomicrobia* at the phylum level. At the genus level, the relative abundance of *Akkermansia, Prevotellaceae, Bacteroides* and *Alloprevotella* was significantly increased after DTE treatment. This study provides multiomics molecular evidence for the intervention effect of DTE on abnormal glucose and lipid metabolism and the application of precise nutritional diet intervention of dark tea extract.

## Introduction

Glucolipid metabolism disorder is a metabolic disorder that seriously threatens human health and affects the quality of life. Glucolipid metabolism disorders mainly take insulin resistance, oxidative stress, chronic inflammation, and intestinal flora imbalance as their core pathologies, resulting in systemic multi-tissue organ dysfunction and multi-system damage, which need to be comprehensively prevented and controlled from the whole body (1). Insulin-related signaling pathways such as IRS/PI3K/AKT/GLUT4, IRS/PI3K/GSK, MAPK/ERK, and PI3K/Akt/GSK play important roles in the development of metabolic syndrome associated with abnormal glucolipid metabolism. In addition, insulin resistance induces heterotopical lipid deposition and reduces mitochondrial function, which in turn leads to storms of inflammatory factors and induces oxidative imbalance in the body. Oxidative stress state destroys antioxidant enzymes, and ultimately leads to abnormal glucolipid metabolism (2, 3). The NF-κB signaling pathway is an important link between inflammation and glucolipid metabolism disorder (4). PPARγ can inhibit the activity of NF-κB DNA and reduce the expression of NF-κB mediated inflammatory mediators, finally exerting the anti-inflammatory effect (5). Concurrently, PPARγ can regulate the expression of various adipokines, inflammatory factors in adipocytes, insulin signal transduction, and regulate the regular balance of insulin and glucolipid metabolism. After investigating the intestinal flora of obese and normal people, it is found that the risk of metabolic diseases such as diabetes and nonalcoholic fatty liver disease will be increased in people with low microbial abundance (6). According to a study on the intestinal flora of 345 Chinese patients with T2DM, the abundance of *Firmicutes* and *Clostridium* in the intestinal flora of patients with diabetes mellitus tended to decrease (7).

Dark tea is one of the six major teas in China and belongs to post-fermented teas. Depending on its origin and different fermentation method, it includes ripe Pu'er Tea, Fuzhuan Brick Tea, Liupao Tea, Qing Brick Tea and Kang Brick Tea (8). Dark tea is rich in bioactive components, such as alkaloids, flavonoids, free amino acids, polyphenols, polysaccharides and volatile compounds, etc. A large number of research reports have confirmed that, dark tea and its extracts had significant effects in regulating abnormal glucolipid metabolism (9, 10). Many studies have shown that dark tea can significantly inhibit the absorption rate of glucose and enhance the insulin resistance (11). A study reported that Qingzhuan tea extracts exerted potent inhibitory effects against α-glucosidase (12). Instant dark tea with high levels of theabrownines showed antioxidant ability and inhibitory activities of α-glucosidase and pancreatic lipase (13). A series of *in vivo* studies also found that dark tea affected abnormal glucose metabolism by decreasing insulin resistance. Fuzhuan Brick Tea significantly antagonized HFD-induced insulin resistance with elevations in serum leptin, TC, TG, LDL-C, blood urea nitrogen, uric acid, and creatinine levels. Furthermore, Fuzhuan brick tea alleviated insulin resistance through down-regulation of SIRP-α expression and activation of the insulin signaling Akt/GLUT4, FoxO1, and mTOR/S6K1 pathways in skeletal muscle (14). Pu'er Tea extract improve the insulin sensitivity and glucose tolerance by increasing the expression of GLUT4 in adipose tissues, maintaining insulin signal transduction and reducing the expression of gluconeogenesis-related genes in the liver (15). Moreover, studies have shown that black tea can improve the oxidation and catabolism of fatty acids, while inhibiting the generation and accumulation of fat. Fuzhuan Brick Tea polysaccharides could up-regulate the expression of genes related to fatty acid decomposition in rats and down-regulate the expression of genes related to lipogenesis (16). A randomized, double-blind, placebo-controlled study showed that consumption of Pu'er Tea extract was associated with improvements to lipid profile, including a mild reduction in cholesterol and the cholesterol: high-density lipoprotein ratio after only 4 weeks, as well as a reduction in triglycerides and very small-density lipoproteins (17). Pu'er Tea also ameliorates hepatic lipid metabolism, inflammation, and insulin resistance in mice with HFD-induced nonalcoholic steatohepatitis, presumably by modulating hepatic IL-6/STAT3 signaling (18). In conclusion, dark tea or its extracts may affect abnormal glucolipid metabolism by reducing blood glucose levels, reducing insulin resistance, affecting the metabolism of fatty acid, and improving the related inflammation factors.

Multi-Omics combines two or more omics research methods, such as genomics, transcriptomics, microbiology, proteomics or metabolomics, to conduct systematic research on biological samples, and integrate and analyze the data of each omics to deeply mine the biological data. In recent years, a large number of studies have unveiled the molecular mechanisms of abnormal glucolipid metabolism using multi-omics technology, as well as precision nutritional intervention based on transcriptomics. Currently, relevant studies have explored the effects of black tea on gene expression regulators and intestinal microecological balance based on multiomic technology. Study on that utilization of microbiological technology, Fuzhuan Brick Tea-induced increase in abundances of beneficial bacteria *Clostridiaceae, Bacteroidales*, and *Lachnospiraceae* and decreases in harmful *Ruminococcaceae, Peptococcaceae, Peptostreptococcaceae*, and *Erysipelotrichaceae* were causal antecedents for Fuzhuan Brick Tea to reduce obesity and improve metabolic disorders (19).

In this study, the extract of dark tea was used to investigate the effects of blood glucose and lipid levels in diabetic mice. Then, RNA-Seq sequencing was performed on the liver of the mice to analyze the gene expression differences in the liver, screen the differentially expressed genes, perform Gene Ontology (GO) functional annotation and enrichment analysis on the differentially expressed genes, and further perform Kyoto Encyclopedia of Genes and Genomes (KEGG) signaling pathway annotation and enrichment analysis on the differential genes. Finally, 16s rDNA sequencing was conducted on mouse droppings to preliminary analyze structure, abundance, diversity, the differential species at the genus level and the significantly differential species of intestinal micro-organisms in mice. This study will provide new foundations and insights for precision nutritional intervention and the action mechanism of dark tea in metabolic syndrome.

## Materials and methods

### Materials and chemicals

Dark tea extract (DTE) was provided by Hunan Tea Industry Group Co. Ltd. (Changsha, China). Metformin hydrochloride (MET) was purchased from Beijing Jingfeng Pharmaceutical Group Co., Ltd. (Beijing, China).

### Animals and models

There were 40 male diabetic db/db mice and 10 C57BL/6 mice (8 weeks old, specific pathogen free grade) were obtained from Hunan Jingda Experimental Animal Co., Ltd. (SCXK (Xiang) 2019-0004) and raised in the barrier environment of Hunan Drug Safety Evaluation Research Center (SYXK (Xiang) 2015-0016). SPF male db/db mice were fasted for 12 h, and their fasting blood glucose was detected. Forty animals with fasting blood glucose higher than 11.1 mmol/L and body weight ranging from 28.9 to 36.0 g were randomly divided into control group, MET group (0.26 g/kg), low-dose DTE group (DTE-L, 0.78 g/kg) and high-dose DTE group (DTE-H, 1.56 g/kg), with 10 animals in each group. Another 10 male C57BL/6 mice of SPF were taken as the normal control group, with blood sugar values of 3.1–7.2 mmol/L and body weight of 19.3 23.1 g. Each dose group was given the corresponding drugs by gavage of 20 ml/kg once a day for 11 weeks, while the normal control group and the model control group were given the same volume by gavage. During the experiment, the weight and blood glucose of animals were monitored regularly every week. At the end of 11th week intervention, the mice were sacrificed by cervical vertebra after ether anesthesia. Then, serum was obtained from mouse eyes after centrifugation. Afterwards, liver and pancreas were collected and stored at-80 for further use.

### Serum parameters analysis

The serum were used to evaluate triglyceride (TG), total cholesterol (CHO), high-density lipoprotein cholesterol (HDL), low-density lipoprotein cholesterol (LDL) (Wako Pure Chemical Industries, Ltd., Chuo-ku, Japan). Blood glucose level was measured using blood glucose test strip (Sinocare Inc Co. Ltd, Changsha, China). Insulin detection kit from Jiangsu MEIMIAN Biological Industry Co., Ltd. (Yancheng, China).

### IRI and histopathology analysis

IRI = fasting blood glucose (mmol/L) × fasting insulin (mIU/L)/22.5. Histological Analysis and Morphometry Part of the liver tissue was fixed with 4% paraformaldehyde. After 24 h, the livers were embedded in paraffin wax. Then, the liver samples were processed using cryostat (CM1950, Leica, Germany) and stained with haematoxylin–eosin (H&E). Finally, these slices were observed using the Olympus light microscope.

### Total RNA extraction and sequencing

The extraction and detection of total RNA of mice in each experimental group were completed by Beijing Novogene Technology Co., Ltd. (Beijing, China). RNA from the total sample was isolated and purified using Trizol (Invitrogen, USA). The quantity and purity of total RNA were then controlled by NanoDrop ND-1000, RNA integrity was tested by Bioanalyzer 2100, and RNA concentration was quantified by agarose electrophoresis. MRNAs with polyadenylation (PolyA) were purified by using oligo (dT) magnetic beads for specific capture, and the captured mRNA was fragmented by using a magnesium ion disruption kit at 94°C for 5–7 min under a high temperature condition, and the obtained fragmented RNA was reverse transcribed into cDNA by reverse transcriptase action. *E. coli* DNA polymerase I and RNase H were used to convert the compound double chains of these DNA and RNA into DNA double chains, and dUTP Solution was added into the two chains to fill the ends of the double-stranded DNA into flat ends and add an A base at each end to enable it to connect with the linker with T base at the end. Their fragment sizes were subsequently screened and purified using oligo(dT) magnetic beads. To be maintained at 95°Cfor 3 min by PCR-predenaturation, 98°C for a total of eight cycles of 15 s each, annealing to 60°C for 15 s, extension at 72°C for 30 s, and finally extension at 72°C for 5 min to form a library with a fragment size of 300 ± 50 bp. Finally, it was double-ended sequenced using Illumina NovaSeq™ 6,000 according to standard operations in sequencing mode PE150.

### qRT-PCR analysis

To measure the expression of *Fabp1, Cyp4a1, Ehhadh, Cyp4a32, Aqp7* and *Me1* in the liver, total RNA of the liver was extracted using Omega total RNA Kit. RNA was reversely transcribed using PrimeScript RT Master Mix. QPCR primers were designed by Biological Engineering Co., Ltd. (Shanghai, China) based on the cDNA sequence. Real-time quantitative reverse transcription PCR (qRT PCR) was performed using SYBR GreenMaster Mix and QuantStudio 3 Flex according to the manufacturer's guidelines. The experiments were performed on a flat plate, three in each group in parallel, and using the 2^−▵▵*Ct*^ method. The reaction procedure was as follows: 95°C for 10 min using a three-step cycle using the Rotor-Gene Q6200 real-time PCR system followed by 40 cycles of 95°C for 10 s, 60°C for 20 s and 72°C for 20 s. The reaction mixture (20 μl) consisted of 2 μl cDNA solution, 10 μl IQTM SYBRR Green Supermix, and 6 μl primers. Three for each group were parallel and the results were averaged. β-actin was used as a reference gene. The comparative cycle threshold method was used to evaluate the relative expression level of genes.

### DNA extraction and 16S gene sequencing

DNA from different samples was extracted using the EZNA tool DNA Kit (D4015, Omega, Inc., USA) according to the manufacturer's instructions. The reagent that was designed to uncover DNA from trace amounts of the sample has been shown to be effective for the preparation of DNA of most bacteria. Nuclear-free water was used for blank. The total DNA was eluted in 50 μL of elution buffer and stored at −80°C until PCR measurement.

### Statistical analysis

Results were expressed as mean ± SD. The significant differences between groups were analyzed by the One-way ANOVA followed Tukey's multiple comparison post-test, and by using the LSD procedure of SPSS 26, and different statistical significance were accepted at *p* < 0.05. The correlation between gut microbiota, serum index and metabolites were analyzed by SPSS statistical program. Statistical significance was set at *p* < 0.05.

## Results

### Effects of DTE on metabolic parameters including serum lipid and blood glucose levels in db/db mice

**Figure 1:**
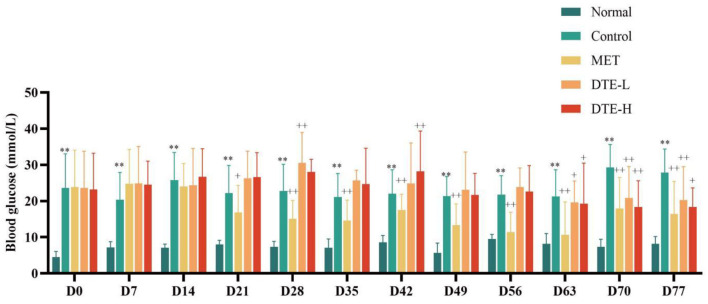
The effect of DTE on fasting blood glucose of db/db diabetic mice. Control, MET, DTE-L, DTE-H vs. Normal, **p < 0.01; MET, DTE-L, DTE-H vs. Control, ^+^*p* < 0.05, ^++^*p* < 0.01.

As shown in Figure 1, compared with the normal group, the fasting blood glucose of mice in the control group was significantly increased (*p* < 0.01). Compared with the control group, there is no significant hypoglycemic effect was observed in the DTE-L and DTE-H group at the levels of D0, D7, D14, D21, D28, D35, D42, D49, and D56. The fasting blood glucose of db/db diabetic mice in the DTE-L and DTE-H group was significantly reduced (*p* < 0.05 or *p* < 0.01) when D63, D70, and D77 were given. Besides, the reducing effects of the DTE-H group of fasting blood glucose in dB/dB diabetic mice were better than the DTE-L group in D49, D56, D63, D70, and D77. The fasting blood glucose of db/db mice in the MET was significantly reduced (*p* < 0.05 or *p* < 0.01) after administration of D21, D28, D35, D42, D49, D56, D63, D70, and D77.

HDL of the DTE-L and DTE-H groups showed an upward trend with no statistical difference with respect to the normal group. The content of TG of the control group, DTE-L group and DTE-H group tended to be decreased, but there was no statistical difference. The CHO level in the DTE-L group and DTE-H group was lower than that that of control group, but there was no statistical difference. Relative to the control group, the LDL level of the MET group increased significantly (*p* < 0.05) (Table 1).

**Table 1:** The effect of DTEon blood lipid levels.

	**TG (mmol/L)**	**CHO (mmol/L)**	**HDL (mmol/L)**	**LDL (mmol/L)**
Normal	0.45 ± 0.19	2.80 ± 0.35	2.25 ± 0.25	0.26 ± 0.12
Control	0.37 ± 0.29	3.38 ± 0.44	2.95 ± 0.45	0.26 ± 0.09
MET	0.72 ± 0.55	4.65 ± 1.54^+^	3.75 ± 0.71	0.61 ± 0.82^+^
DTE-L	0.38 ± 0.12	3.15 ± 1.49	3.20 ± 4.50	0.23 ± 0.17
DTE-H	0.32 ± 0.27	3.24 ± 1.61	2.99 ± 1.14	0.38 ± 0.25

MET, DTE-L, DTE-H vs. Control,

+ *p* < 0.05.

### Effect of DTE on serum insulin level and IRI of db/db mice

Compared with the normal group, serum insulin of db/db diabetic mice in the control group tended to decrease, and the IRI was significantly increased (*p* < 0.01). Compared with the control group, the serum insulin levels of db/db diabetic mice in the DTE-H group were significantly increased. The IRI of db/db diabetic mice in the MET group was decreased significantly (*p* < 0.01) (Table 2).

**Table 2:** The effect of DTE on insulin and insulin resistance index (IRI).

	**Insulin (mIU/L)**	**IRI**
Normal	48.08 ± 2.74	16.13 ± 3.96
Control	43.80 ± 4.00	47.57 ± 15.89^**^
MET	48.56 ± 2.76	26.87 ± 14.46^++^
DTE-L	46.25 ± 3.19	43.84 ± 11.20
DTE-H	48.63 ± 4.72	40.31 ± 12.62

Control, MET, DTE-L, DTE-H vs. Normal,

** *p* < 0.01; MET, DTE-L, DTE-H vs. Control,

++ *p* < 0.01.

### Effect of DTE on morphology of pancreas tissue in mice

**Figure 2:**
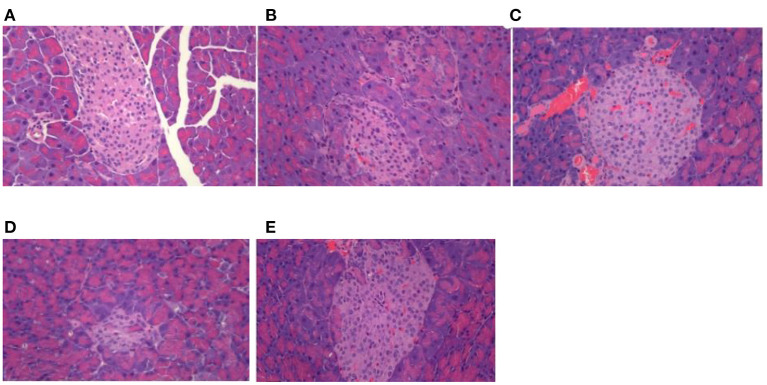
Effect of DTE on morphology of pancreas tissue in mice. **(A)** Normal group; **(B)** Control group; **(C)** MET group; **(D)** DTE-L group; **(E)** DTE-H group.

As shown in Figure 2, the islets were spherical cell masses composed of endocrine cells, and each islet was composed of many cells, with a clear islet boundary in the normal control group. Compared to the normal group, the pathological score of pancreatic tissue hyperplasia in db/db diabetic mice was significantly increased (*p* < 0.01). In the control group, the islets of langerhans were seriously damaged, and the number of islets was significantly reduced, with the result that the islets became smaller, and the boundary was fuzzy. The number of β cells in the islets was significantly reduced, which was disorganized, with partial cell necrosis and unclear cell boundary. Compared to the control group, the pathological scores of pancreatic tissues in db/db diabetic mice in the MET group tended to decrease, but there was no statistical difference. The DTE-H group and MET group could slightly improve the damage of pancreatic tissue, vague islet boundary, and disordered arrangement of islet β cells in diabetic mice.

### Effect of DTE on gene expression in liver of db/db mice

**Figure 3:**
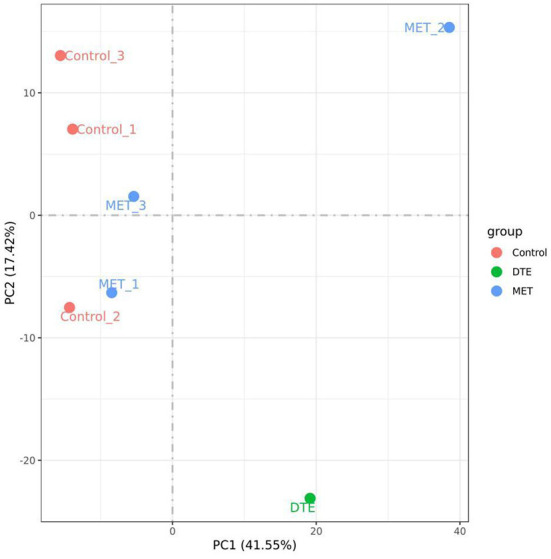
The PCA of the samples. The experimental data showed significant differences between the DTE group, MET group, and control group.

Data quality control required Q20> 90%, Q30> 85%, and GC content of 40–50% after filtration to indicate no abnormalities during sequencing. The minimum values of Q20 and Q30 for the samples were 96.75 and 91.30%, respectively, and the GC content was within the range of 46.49–49.86%, indicating that the sequencing quality was preferable. Principal component analysis (PCA) was also frequently used to assess inter-group differences and sample repetition within groups. Regarding the PCA data of the DTE group, we previously performed three sets of DTE, but only one set of experimental data was valid, and the experimental data showed significant differences between the DTE group, MET group, and control group. As shown in Figure 3, PC1 accounted for 41.55%, and it could significantly distinguish between the control group and other treatment groups, indicating that the other treatment groups had significant differences in liver transcriptional expression. PC2 accounted for 17.42%, significantly differentiating the MET group from the DTE group, indicating that DTE was able to regulate mouse gene expression at the transcriptome level.

**Figure 4:**
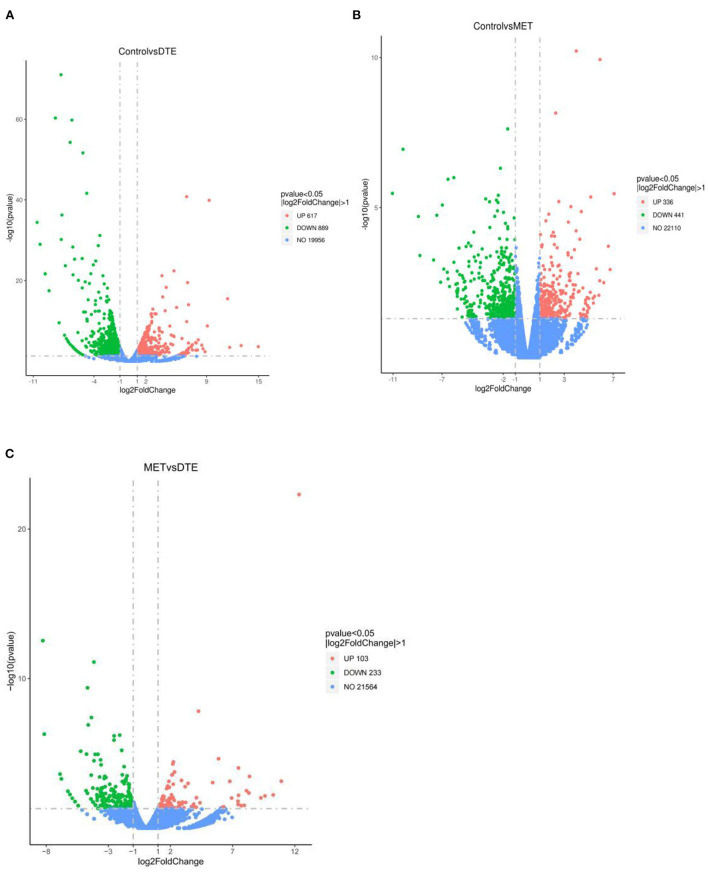
Volcanic map of difference gene expression between groups. **(A)** Control vs. DTE; **(B)** Control vs. MET; **(C)** MET vs. DTE.

The data of gene transcriptional expression levels of the control, MET and DTE group obtained by RNA-Seq sequencing are shown in Figure 4. Compare to the Control group, DTE group had 1,506 differential genes, including 617 up-regulated genes and 889 down-regulated genes; Compare to the Control group, there were 777 differential genes in the MET group, including 441 down-regulated genes and 336 up-regulated genes; Compare to the DTE group, there were 336 differential genes in the MET group, including 233 down-regulated genes and 103 up-regulated genes.

**Figure 5:**
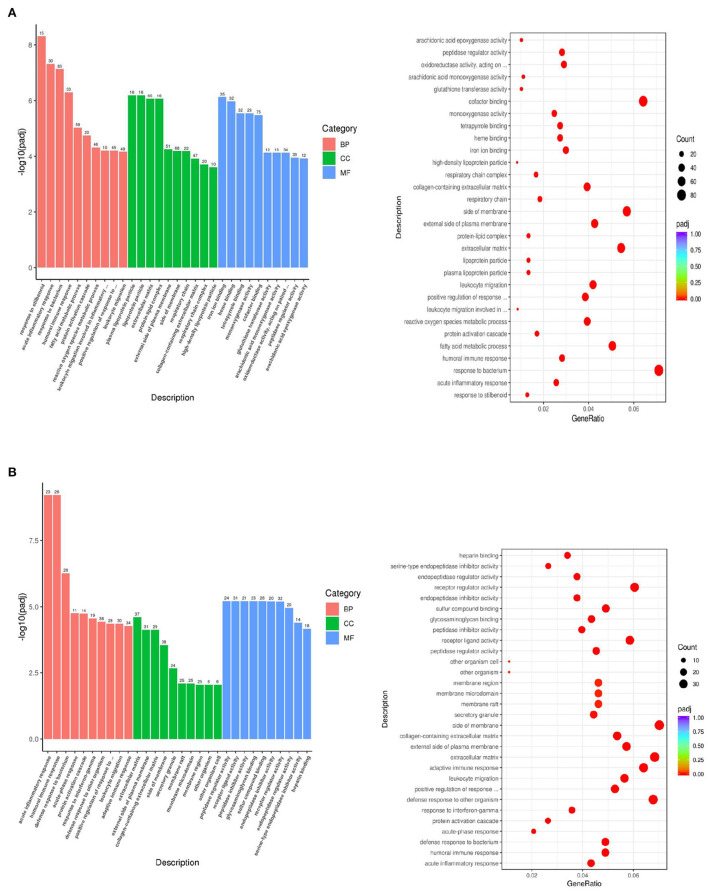
GO function analysis of difference gene expression. **(A)** Control vs. DTE; **(B)** Control vs. MET.

Compare to the DTE group, the GO functional classification and enrichment analysis of differentially expressed genes in the control group were shown in Figure 5A. A total of 6,574 items were screened out, and a total of 423 items with significant statistical significance were screened out, including 294 items concerning biological processes (BP), 49 items concerning cellular components (CC) and 80 items concerning molecular functional processes (MF). In biological processes, the most significant 10 items were: response to stilbenoid, acute inflammatory response, response to bacterium, humoral immune response, fatty acid metabolic process, protein activation cascade, reactive oxygen species metabolic process, leukocyte migration involved in inflammatory response, positive regulation of response to external stimulu, and leukocyte migration. In cellular component, the most significant 10 items were: plasma lipoprotein particle, lipoprotein particle, extracellular matrix, protein-lipid complex, external side of plasma membrane, side of membrane, respiratory chain, collagen-containing extracellular matrix, respiratory chain complex, and high-density lipoprotein particle. In the molecular functional process, the most significant 10 items were: iron ion binding, heme binding, tetrapyrrole binding, monooxygenase activity, cofactor binding, glutathione transferase activity, arachidonic acid monooxygenase activity, oxidoreductase activity, peptidase regulator activity, and arachidonic acid epoxygenase activity. The biological effects of DTE are mainly concentrated on anti-inflammation, anti-oxidation, anti-bacteria, and fatty acid metabolism. In terms of cell composition, DTE mainly affects its lipoprotein and respiratory chain. In terms of molecular functional process, DTE mainly affects the activity of redox-related enzymes, iron ion binding and glutathione transferase, etc. Thus, DTE showed a regulatory effect on lipid metabolism, inflammation, and redox as compared with the control group.

Compare to the MET group, the GO functional classification and enrichment analysis of differentially expressed genes in the control group are shown in Figure 5B. A total of 5,168 items were screened out, and a total of 248 items with significant statistical significance were screened out, including 191 items on biological process, 24 items on cellular component and 33 items on molecular functional process. In biological process, the most 10 significant items are: acute infectious response, humoral immune response, defense response to bacterium, acute-phase response, protein activation cascade, response to interferon-gamma, defense response to other organism, positive regulation of response to external stimulus, leukocyte migration, and adaptive immune response. In cellular component, the most 10 significant items are: extracellular matrix, externally side of plasma membrane, collagen-containing extracellular matrix, side of membrane, secretory grand, membrane raft, membrane microdomain, membrane region, other organism cell, and other organism cell. In items of molecular function, the most 10 significant entries were: peptidase regulator activity, receptor ligand activity, peptidase inhibitor activity, glycosaminoglycan binding, sulfur compound binding, endopeptidase inhibitor activity, receptor regulator activity, endopeptidase regulator activity, serine-type endopeptidase inhibitor activity, and heparin binding.

### KEGG pathway analysis

**Figure 6:**
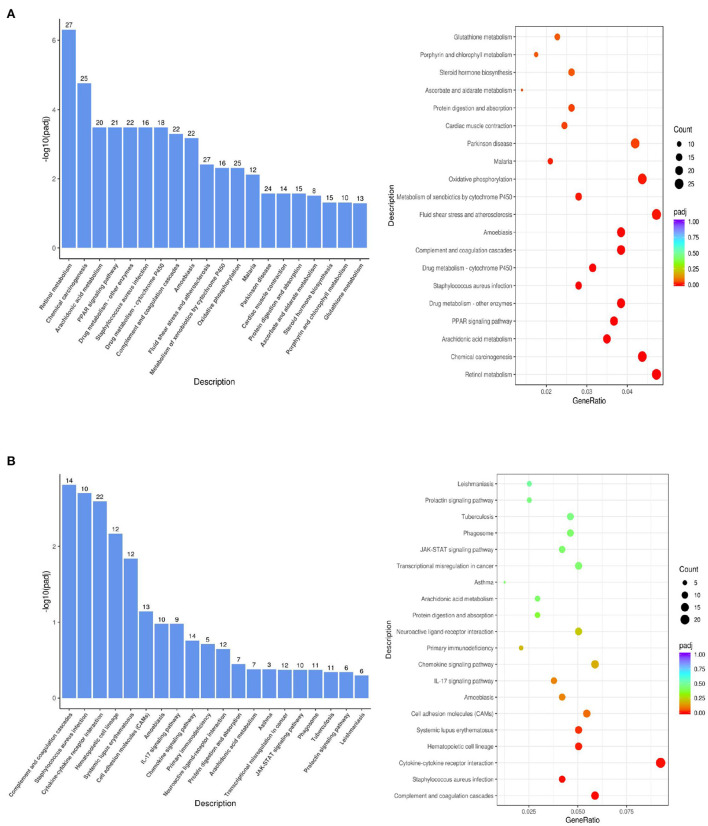
KEGG pathway analysis of the difference gene expression. **(A)** DTE vs. Control; **(B)** MET vs. Control.

As shown in Figure 6A, the KEGG biological pathways significantly enriched in the DTE group were as follows: Retinol metabolism, Chemical carcinogenesis, Arachidonic acid metabolism, PPAR signaling pathway, Drug metabolism enzymes, Staphylococcus aureus infection, Drug metabolism-cytochrome P450, Complement and coagulation cascades, Amoebiasis, atherosclerosis, Glutathione metabolism, and Oxidative phosphorylation. As shown in Figure 6B, the KEGG biological pathways significantly enriched in the MET group were as follows: Complement and coagulation cascades, *Staphylococcus aureus* infection, Cytokine-cytokine receptor interaction, Hematopoietic cell lineage, Systemic lupus erythematosus, Cell adhesion molecule, Amoebiasis, IL-17 signaling pathway, Chemokine signaling pathway, Glutathione metabolism, Primary immunodeficiency, Arachidonic acid metabolism.

**Figure 7:**
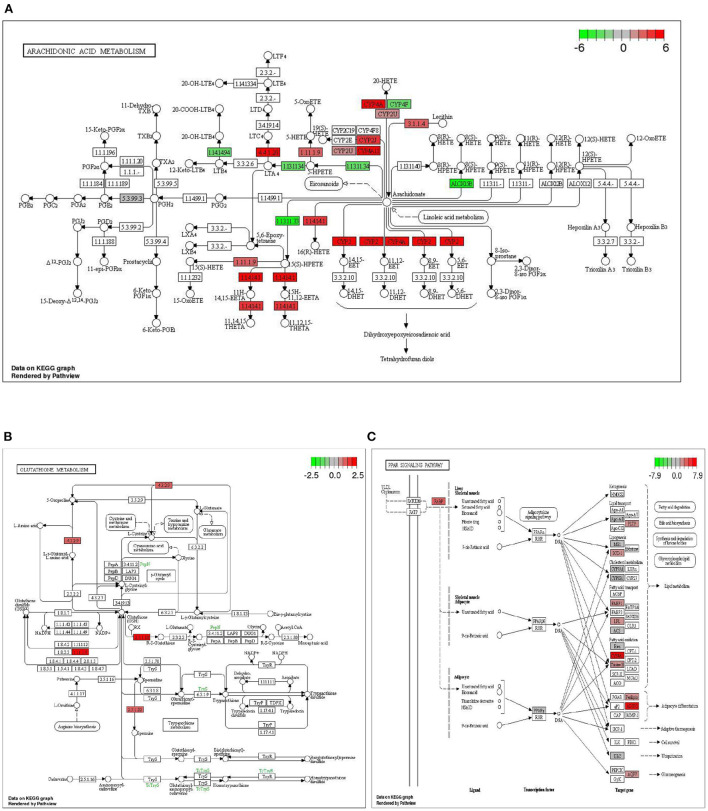
Significantly enriched signaling pathway by DTE. **(A)** Effect of DTE on Arachidonic acid metabolism pathway; **(B)** Effect of DTE on Glutathione metabolism pathway; **(C)** Effect of DTE on PPAR signaling pathway.

In the comparison between DTE group and MET group, we found that two common signal pathways: Arachidonic acid metabolism pathway and Glutathione metabolism were significantly enriched. As shown in Figure 7A, in the arachidonic acid metabolism signaling pathway, the cytochrome P450 (CYPs) such as *CYP2, CYP4A*, and *CYP4A11* are significantly upregulated. As shown in Figure 7B, in the glutathione metabolic pathway, *Cyp4f18, Cyp2b13, Ltc4s, Pla2g4f* , *Alox15*, and *Cyp2c40* genes were significantly upregulated. In the comparison between the DTE group and the control group, we found that the PPAR signaling pathway also exhibited significant enrichment. As shown in Figure 7C, in the PPAR signaling pathway, the *Cyp4a12a, Cyp4a1, Acaa1b, Cyp4a10, Pltp, Plin4, Scd1, Cyp4a1, Apoa2, Pparg, Lpl, Cyp8b1, Fabp2, Adipoq, Cyp7a1, Me1, Cyp4a32, Ubc/Acsl1* genes were up-regulated, and the *Ehhadh, Fabp1, FABP3, FABP4* and *Aqp7* genes were down-regulated, all of which were closely related to glycolipid metabolism.

### qRT-PCR analysis

**Figure 8:**
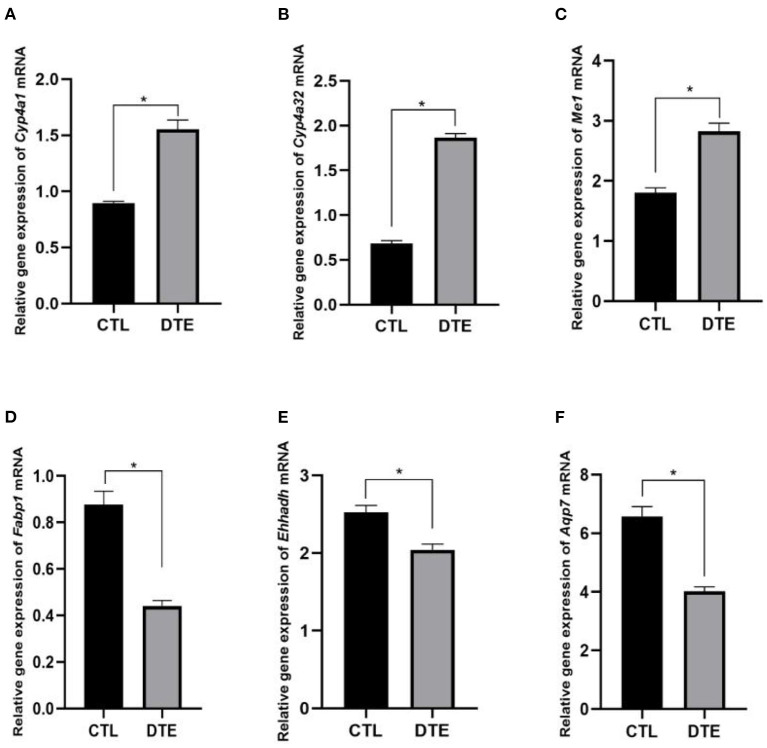
Effects of DTE on expression of PPAR signaling pathway related genes by qRT-PCR. **(A)** *Cyp4a*; **(B)** *Cyp4a32*; **(C)** *Me1*; **(D)** *Fabp1*; **(E)** *Ehhadh*; **(F)** *Aqp7*; DTE vs. Control, *p < 0.05; **p < 0.01.

To verify the effect of DTE on PPAR signaling pathway, qRT-PCR technology was used to analyze the genes related to glucolipid metabolism of *Cyp4a1, Cyp4a32, Me1, Fabp1, Ehhadh*, and *Aqp7* in PPAR signaling pathway. The results of qRT-PCR were shown in Figure 8. Compared with the control group, the expressions of *Cyp4a1, Cyp4a32*, and *Me1* genes involved in glycolipid metabolism were significantly up-regulated in DTE group, and the expressions of *Fabp1, Ehhadh*, and *Aqp7* were significantly decreased (*p* < 0.05), suggesting that the extract of DTE could significantly activate key genes of PPAR signaling pathway, and the transcriptome sequencing result was credible.

### Effects of DTE on composition of gut microbiota

As shown in Table 3, compare to the normal group, the community diversity, total number of species and abundance in the control, MET and DTE groups were decreased. Compare to the control group, the community diversity, total number of species and abundance in the MET group were decreased, while the community diversity in the DTE group was decreased, but the total number of species and abundance were increased without significant difference (*p* > 0.05). Compare to the MET group, the community diversity of the DTE group was decreased, and the total number and abundance of species were increased, but there was no significant difference (*p* > 0.05).

**Table 3:** Effect of DTE on intestinal microbial diversity db/db diabetic mice.

**Sample name**	**Observed_** **species**	**Shannon**	**Chao 1**	**ACE**
Normal	473.67 ± 25.15	5.89 ± 0.36	536.15 ± 47.53	535.36 ± 41.24
Control	415 ± 62.04	5.45 ± 0.25	461.54 ± 71.88	470.8 ± 70.52
MET	304.67 ± 13.64	4.56 ± 0.63	348.44 ± 28.88	358.89 ± 33
DTE	451 ± 25.83	4.420 ± 0.29	493.873 ± 36.47	503.556 ± 40.52

Compare to the normal group, the community diversity, total number of species and abundance in the control, MET and DTE groups were decreased.

**Figure 9:**
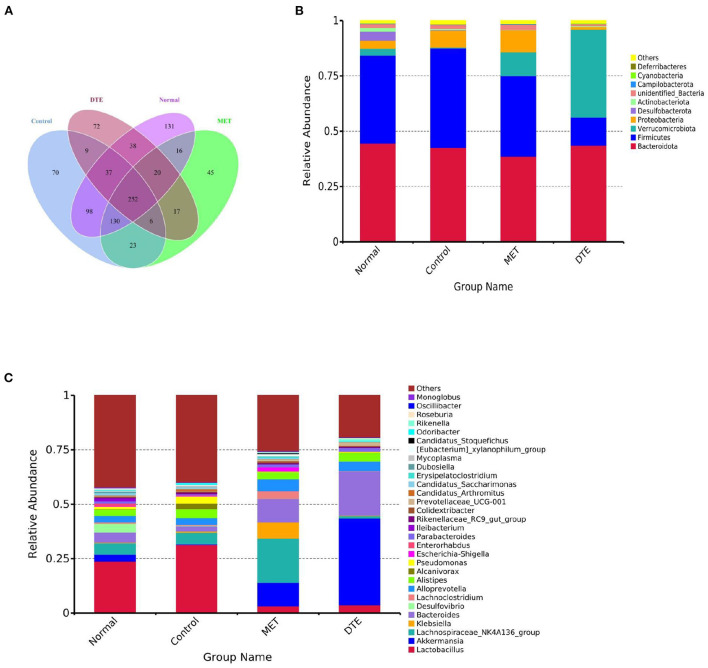
Effect of DTE on intestinal microbial community of diabetic mice. **(A)** Venn diagram based on OTU; **(B)** Effect of DTE on intestinal microbial community of diabetic mice at phylum species level; **(C)** Effect of DTE on intestinal microbial community of diabetic mice at genus level.

To study how the intestinal microflora of mice changed, we compared different groups of OTUs (Figure 9). There are 131 differences in normal group, 70 differences in control group, 45 differences in MET group and 72 differences in DTE group, which indicates that the abundance of intestinal microorganisms in each group is different. In addition, there are 252 identical OTUs in the four comparison groups, and the OTUs in the control group are more abundant than those in other groups.

At the phylum level, as shown in Figure 9B, the relative abundance of *Bacteroidetes* and *Firmicutes* is higher in MET group and DTE group. Among the four groups, the relatively abundant ones are: *Bacteroidetes, Firmicutes, Verrucomicrobia, Desulfobacterota, Proteobacteria, Actinobacteriota, Campilobacterota, Cyanobacteria*, and *Deferribacter*. In normal group, control group, MET group and DTE group, the ratio of F/B was 0.89, 1.05, 0.94, 0.48, and 0.29, respectively. Compare to the normal group, the relative abundance of *Proteobacteria* in control group increased. In addition, compared with control group, DTE group significantly increased the relative abundance of *Verrucomicrobia*.

At the genus level, as shown in Figure 9C, the relative abundance of beneficial bacteria such as *Bacteroide, Akkermansia*, and *Alloprevotella* was decreased in the control group. The MET group significantly increased the relative abundance of *Lachnospiraceae NK4A136, Alloprevotella*, and *Escherichia shigella*. The DTE group significantly increased the relative abundance of *Akkermansia, Prevotella, Bacteroides*, and *Alloprevotella*.

## Discussion

With the changes in diet structure, lifestyle and the increasingly serious aging trend, the incidence of metabolic syndrome, with glycolipid metabolic disorders as the core, is showing a rapidly rising trend worldwide (20). How to efficiently restore mitochondrial function, regulate oxidative stress and inflammatory signaling pathways is the goal of improving disorders of glucolipidic metabolism (21). The natural food active ingredient has remarkable effect on regulate glycolipid metabolism disorder. Cellular, animal, and clinical studies have shown that dark tea has a variety of physiological functions and health benefits, including anti-oxidant (22), anti-inflammatory (23), anti-cancer (24), anti-diabetes (25), prevent cardiovascular disease (26), and regulation of intestinal microbiota (27). However, effects of DTE on glucolipid metabolism by multi-omic strategy have been rarely studied. Thus, in this study, 16S rDNA and metabonomic approach were applied to comprehensively investigate the effects of DTE on glucolipid metabolism. Serum parameters indicated that DTE could regulate blood glucose and lipid levels. 16S rDNA sequencing and metabolomics showed significant effects on the composition of the intestinal microbiota and liver metabolites.

Our study showed that DTE may ameliorate metabolic disorders in db/db diabetic mice by significantly lowering blood glucose, serum insulin level, TC, TG, LDL, and lightly improve the islet damage. Dark tea has been reported to play an important role in reducing blood lipid and blood glucose. Fuzhuan brick tea water extracts could significantly suppress the increase of body weight and accumulation of adipose tissue, and reduced the level of serum triacylglycerol, total cholesterol and low-density lipoprotein (LDL) cholesterol in obese rats fed a high-fat diet (28). Qing Brick Tea exerted remarkable functions on decreasing the level of serum triglyceride and preventing hepatic fat accumulation (29). Pu'er Tea was found to have anti-hyperglycemic effects *via* inhibition on alpha-amylase and alpha-glucosidase (30). The above studies are consistent with our experimental results that DTE can regulate blood glucose and blood lipid.

RNA-Seq technique was used to preliminarily analyze the gene expression of mouse liver, and the potential differential metabolites and related metabolic pathways were analyzed from the perspective of metabonomics, which could reveal the effect of DTE on metabolic reaction of life system from the overall and dynamic perspective. Through GO functional classification and enrichment analysis, we found that DTE effects on biological processes mainly focused on anti-inflammatory, antioxidant, antibacterial and fatty acid metabolism. In terms of cell components, DTE mainly affects lipoprotein and respiratory chain. In terms of molecular functional process, DTE mainly affects its redox-related enzyme activity, iron binding and glutathione transferase. MET has been recognized as an effective drug to regulate glycolipid metabolism and reduce blood glucose (31). Compared with MET group, DTE group has some similarities in BP, CC, and MF. We speculate that DTE may have potential biological functions such as regulating glucose and lipid metabolism and improving inflammatory response.

By analyzing the enrichment results of KEGG pathway, we found that three typical signal pathways: Arachidonic acid metabolism pathway, Glutathione metabolism and PPAR pathway were significantly enriched. Arachidonic acid plays an important role in blood, liver, muscle and other organ systems as phospholipid-bound structural lipid. Cytochrome P450 (CYP) is a kind of protein with iron porphyrin as the auxiliary group, also known as monooxygenase, which mainly exists in the endoplasmic reticulum of liver cells. This enzyme can promote the metabolism of drugs, so it is also known as liver drug enzyme. Arachidonic acid is a bioactive substance of many circulating eicosanoic acid derivatives, such as prostaglandin E2 (PGE2), prostacyclin (PGI2), thromboxane A2 (TXA2), leukotrienes and C4 (LTC4), which plays an important role in regulating lipid protein metabolism, vascular elasticity, leukocyte function and platelet activation (32). In the signal pathway of arachidonic acid metabolism, CYP such as *CYP2, CYP4A, CYP4A11* are significantly up-regulated. It was found that the extract of Chenopodium album can reduce the activity of cytochrome P4502E1 (*CYP2E1*), enhance the activity of catalase and improve the histological structure of rat liver to reduce the oxidative stress (33). We speculate that DTE may intervene the abnormal metabolism of glucose and lipid in the liver of diabetic mice through arachidonic acid metabolism.

Glutathione (GSH) is the most abundant antioxidant and a major detoxification agent in cells. It is synthesized through two-enzyme reaction catalyzed by glutamate cysteine ligase and glutathione synthetase, and its level is well regulated in response to redox change. Accumulating evidence suggests that GSH may play important roles in cell signaling (34). As shown in the effect of DTE on the glutathione metabolic pathway, *Cyp4f18, Cyp2b13, Ltc4s, Pla2g4f* , *Alox15*, and *Cyp2c40* genes were significantly up-regulated. We speculated that DTE might intervene in glucolipid metabolism by regulating oxidative stress through the glutathione metabolic pathway.

PPAR is a core factor in the anti-inflammatory response pathway and participates in the formation of inflammatory and metabolic signal regulatory networks by different pathways, thus significantly affecting the regulation of glucolipid metabolism. PPARγ also can reduce the expression of NF-κB-mediated inflammatory mediators and exert its anti-inflammatory effect (35). PPARγ regulate the expression of many fat and inflammatory factors such as TNFα and regulate the glucolipid metabolism process (36). In the PPAR signaling pathway, *Cyp4a12a, Cyp4a1, Acaa1b, Cyp4a10, Pltp, Plin4, Scd1, Cyp4a1, Apoa2, Pparg, Lp*l, *Cyp8b1, Fabp2, Adipoq, Cyp7a1, Me1, Cyp4a32*, and *Ubc/Acsl1* genes were up-regulated, and *Ehhadh, Fabp1, FABP3, FABP4*, and *Aqp7* genes were down-regulated, all of which were closely related to glycolipid metabolism. The increased expression of *FABP1* is mainly related to the occurrence of insulin resistance and the promotion of fatty acid transport (37). *AQP7* also showed a significant correlation with glucolipid metabolism indicators (38). Since DTE may significantly up-regulate the genes expression of PPAR signaling pathway, we speculate that DTE may have the potential to regulate inflammatory factors, glycolipid metabolism, and promote fatty acid transport.

Intestinal microflora plays an important role in host health, the change of intestinal microflora may induce intestinal oxidative stress, and lead to glycolipid metabolism disorder (39). The ratio of *Firmicutes/Bacteroidetes* is widely believed to be important for maintaining the homeostasis of the intestinal environment. Reduction of the F/B value leads to T2D (40). After treatment with DTE, the value of F/B was decreased, indicating DTE positively regulated the gut microbiota. *Bacteroidetes, Firmicutes, Proteobacteria* and *Verrucomicrobia* can regulate glycolipid metabolism and improve insulin resistance (40). After treatment with DTE, the relative abundance of *Proteobacteria* was decreased, and the relative abundance of *Verrucomicrobia* was increased. These results indicated that DTE could regulate the structural composition of the intestinal flora to improve the intestinal environment and reduce the risks of diabetes and obesity.

At the genus level, treatment with DTE significantly increased the relative abundance of *Akkermansia, Prevotellaceae, Bacteroides*, and *Alloprevotella*. *Akkermansia* can regulate immune response, lipid metabolism, inhibit inflammation, and improve the disorder of intestinal flora. The decrease in abundance is closely related to the development of inflammatory intestinal diseases, T2D and cardiovascular diseases (41). As shown in Figure 9C, the proportion of *Akkermansia* in control group is 0.34%, and that in DTE group is 35.76%. The results showed that DTE could significantly increase the relative abundance of *Akkermansia*, improve the intestinal microenvironment, and then regulate the abnormal of glucose and lipid metabolism.

## Conclusion

In the present study, multi-omics precision nutrition method was applied to carry out multi-dimensional health big data detection and analysis at the animal level from the aspects of the microbiome and RNA sequencing. We found that DTE could significantly reduce the blood glucose in db/db diabetic mice, improve the blood lipid to a certain extent, slightly repair the pancreatic injury, and promote the insulin secretion. According to GO analysis, DTE mainly affected the inflammation, fatty acid metabolism, and redox-related enzyme activities. 16S rDNA sequencing showed that DTE significantly reduced the ratio of F/B and increased the relative abundances of *Akkermansia, Prevotellaceae, Bacteroides*, and *Alloprevotella*. Besides, real-time qPCR was performed to verify the regulatory effect of DTE on PPAR signaling pathway, which provides us with direct and comprehensive molecular evidence for the beneficial effect of DTE on metabolic disorders.

## Data availability statement

The data presented in the study are deposited in the GSA repository: https://ngdc.cncb.ac.cn/gsub/, accession number CRA007923.

## Ethics statement

The animal study was reviewed and approved by Hunan Drug Safety Evaluation Research Center.

## Author contributions

CL and MH wrote the original draft. CL, MH, and CW performed the experiments and collected the samples. CL, YY, and XW contributed to methodology and data analysis. MS and CZ participated in supervision. CL, MS, and CZ contributed to project administration and conceptualization, writing, reviewing, and editing. All authors listed have made a substantial, direct, and intellectual contribution to the work and approved it for publication.

## Funding

This work was partially supported by the National Key Research and Development Program of China (2019YFC1604903) and the Hunan Province Innovative Postdoctoral Project (2021RC2080).

## Conflict of interest

The authors declare that the research was conducted in the absence of any commercial or financial relationships that could be construed as a potential conflict of interest.

## Publisher's note

All claims expressed in this article are solely those of the authors and do not necessarily represent those of their affiliated organizations, or those of the publisher, the editors and the reviewers. Any product that may be evaluated in this article, or claim that may be made by its manufacturer, is not guaranteed or endorsed by the publisher.
